# Is Ticagrelor‐Related Dyspnea a Liability or a Biomarker of Response?

**DOI:** 10.1002/clc.70258

**Published:** 2026-01-11

**Authors:** Abdülmelik Birgün, Abdullah Sarıhan, Macit Kalçık

**Affiliations:** ^1^ Department of Cardiology, Erol Olçok Education and Research Hospital Hitit University Corum Turkey; ^2^ Depertment of Cardiology, Faculty of Medicine Hitit University Çorum Turkey

**Keywords:** clinical decision‐making, CYP3A5, dyspnea, pharmacogenetics, ticagrelor

Dear Editor,

Zhang et al. report a statistically robust association between the CYP3A5 rs776746 polymorphism and ticagrelor‐related dyspnea in patients with acute coronary syndrome, proposing this variant as a candidate biomarker for individualized antiplatelet therapy [1]. While the genetic signal itself is convincing, the broader clinical interpretation of this finding warrants a more cautious and contextualized discussion.

A central issue is the implicit assumption that ticagrelor‐related dyspnea represents an adverse effect that necessarily undermines treatment success. Accumulating evidence suggests that ticagrelor‐induced dyspnea is frequently mild, transient, and poorly correlated with objective respiratory dysfunction, while ticagrelor continuation is often associated with superior ischemic protection [2]. Framing dyspnea primarily as a safety liability may therefore overemphasize tolerability at the expense of long‐term cardiovascular benefit.

Relatedly, the manuscript risks conflating biological association with clinical actionability. Identification of a genotype linked to dyspnea does not automatically justify preemptive drug switching, particularly in the absence of evidence that genotype‐guided de‐escalation preserves ischemic efficacy. Prior pharmacogenetic experiences in antiplatelet therapy have shown that altering treatment based solely on surrogate markers may inadvertently increase thrombotic risk [3].

Another underexplored dimension is the competing mechanistic interpretation of dyspnea as a pharmacodynamic signature rather than a toxic effect. Ticagrelor‐induced increases in adenosine signaling have been associated not only with dyspnea but also with cardioprotective and anti‐inflammatory effects. From this perspective, dyspnea may identify patients with heightened biological responsiveness rather than impaired drug tolerance [4]. Genetic stratification that selectively removes such patients from ticagrelor therapy could paradoxically exclude those deriving the greatest benefit.

Finally, the proposal of routine CYP3A5 genotyping raises broader questions about feasibility and proportionality. Current guidelines emphasize simplicity, timeliness, and net clinical benefit in acute coronary syndrome management. Introducing genetic testing for a largely non‐life‐threatening symptom risks increasing complexity without clear outcome‐level gains [5]. Precision medicine should refine decision‐making, not fragment it.

In this context, the findings by Zhang et al. are best viewed as hypothesis‐generating, highlighting the biological heterogeneity of ticagrelor response rather than defining an immediate clinical algorithm. Future studies should focus on whether dyspnea‐ or genotype‐guided strategies meaningfully improve patient‐centered outcomes while preserving ischemic protection.

## Conflicts of Interest

The authors declare no conflicts of interest.

## Data Availability

Data sharing is not applicable to this article as no data sets were generated or analyzed during the current study.
